# Copy number polymorphisms near SLC2A9 are associated with serum uric acid concentrations

**DOI:** 10.1186/1471-2156-15-81

**Published:** 2014-07-09

**Authors:** Robert B Scharpf, Lynn Mireles, Qiong Yang, Anna Köttgen, Ingo Ruczinski, Katalin Susztak, Eitan Halper-Stromberg, Adrienne Tin, Stephen Cristiano, Aravinda Chakravarti, Eric Boerwinkle, Caroline S Fox11, Josef Coresh, Wen Hong Linda Kao

**Affiliations:** 1 550 N. Broadway, Suite 1101, Department of Oncology, Johns Hopkins School of Medicine, Baltimore, Maryland 21205, USA; 2 Department of Epidemiology, Johns Hopkins School of Public Health, Baltimore, Maryland, USA; 3 Department of Biostatistics, Boston University School of Public Health, Boston, Massachusetts, USA; 4 Department of Medicine IV, University Hospital Freiburg, Freiburg im Breisgau, Germany; 5 Department of Biostatistics, Johns Hopkins School of Public Health, Baltimore, Maryland, USA; 6 Renal Electrolyte and Hypertension Division, Perelman School of Medicine, University of Pennsylvania, Philadelphia PA, USA; 7 Computational Biosciences Program, University of Colorado, Denver, Aurora, Colorado, USA; 8 Department of Biostatistics, Johns Hopkins School of Public Health, Baltimore, Maryland, USA; 9 Department of Medicine, Johns Hopkins School of Medicine, Baltimore, Maryland, USA; 10 IMM Center for Human Genetics, University of Texas School of Public Health, Houston, Texas, USA; 11 Laboratory for Metabolic and Population Health, National Heart Lung and Blood Institute, National Institutes of Health, Framingham, Massachusetts, USA

**Keywords:** Copy number polymorphism, Hyperuricemia, Genomewide association study

## Abstract

**Background:**

Hyperuricemia is associated with multiple diseases, including gout, cardiovascular disease, and renal disease. Serum urate is highly heritable, yet association studies of single nucleotide polymorphisms (SNPs) and serum uric acid explain a small fraction of the heritability. Whether copy number polymorphisms (CNPs) contribute to uric acid levels is unknown.

**Results:**

We assessed copy number on a genome-wide scale among 8,411 individuals of European ancestry (EA) who participated in the Atherosclerosis Risk in Communities (ARIC) study. CNPs upstream of the urate transporter *SLC2A9* on chromosome 4p16.1 are associated with uric acid ($\chi_{2\text{df}}^{2}=3545$, *p*=3.19×10^-23^). Effect sizes, expressed as the percentage change in uric acid per deleted copy, are most pronounced among women (_3.97_4.93_5.87_ [ _2.5_50_97.5_ denoting percentiles], *p*=4.57×10^-23^) and independent of previously reported SNPs in *SLC2A9* as assessed by SNP and CNP regression models and the phasing SNP and CNP haplotypes ($\chi_{2\text{df}}^{2}=3190,\;p=7.23\times 10^{-08}$). Our finding is replicated in the Framingham Heart Study (FHS), where the effect size estimated from 4,089 women is comparable to ARIC in direction and magnitude (_1.41_4.70_7.88_, *p*=5.46×10^-03^).

**Conclusions:**

This is the first study to characterize CNPs in ARIC and the first genome-wide analysis of CNPs and uric acid. Our findings suggests a novel, non-coding regulatory mechanism for *SLC2A9*-mediated modulation of serum uric acid, and detail a bioinformatic approach for assessing the contribution of CNPs to heritable traits in large population-based studies where technical sources of variation are substantial.

## Background

Serum uric acid levels are highly heritable and associated with several diseases, including gout, hypertension, and cardiovascular disease [1-4]. Genome-wide association studies have identified several single nucleotide polymorphisms (SNPs) that are strongly associated with uric acid levels [5-10], but a large proportion of the heritability of uric acid is unexplained by common SNPs. While variation of DNA copy number has been implicated in many heritable diseases, there has been no association studies of copy number polymorphisms (CNPs) and serum uric acid levels on a genome-wide level.

High-throughput platforms used to genotype SNPs are useful for copy number estimation, though additional steps are required to reduce technical artifacts that are prevalent in studies of copy number. Estimates of the relative copy number (log R ratios) and B allele frequencies measured at each marker on the array are mutually informative for the latent copy number [11]. Various hidden Markov model (HMM) implementations integrate the log R ratios and B allele frequencies to infer copy number [12-19]. Copy number estimation is challenging, in part, due to technical artifacts that contribute to false positives. Among the most common artifacts are *genomic waves*[20,21], an autocorrelation of the marker-level estimates when plotted against physical position, and *batch effects*, differences between groups of samples arising from technical sources of variation such as sample preparation, reagents, and laboratory personnel [22-24]. Approaches to reduce wave and batch artifacts include models for adjusting log R ratios by the GC composition of the local sequence as in [21] and surrogates of batch such as chemistry plate in association models when confounding between batch and phenotype is incomplete.

Here, we implement a HMM to infer integer copy number from B allele frequencies and wave-corrected log R ratios obtained from 8,411 ARIC participants of European ancestry assayed on Affymetrix 6.0 arrays. We evaluate the association between CNPs and uric acid concentrations through mixed effects regression models that adjust for available clinical risk factors as well as technical covariates such as chemistry plate and study center. For loci reaching genome-wide significance, we replicate our findings in the Framingham Heart Study (FHS). In addition, we assess whether statistically significant associations among EA participants persist in a smaller cohort of 3,392 African Americans in ARIC. Finally, we establish the independence of the relationship between copy number and uric acid concentrations from genome-wide significant SNP associations among ARIC EA participants.

## Results and discussion

Among 8,411 ARIC samples of European ancestry passing SNP and copy number metrics for quality control (see Methods), 47 percent are male and the mean BMI, uric acid concentration, and age are 27 kg/*m*^2^, 5.9 mg/dL, and 54 years, respectively.

Copy number estimates 0-4 were obtained from a HMM [14]. In this population, the median number of deletions and duplications is 55, and the median cumulative number of bases spanned by copy number variants (CNVs) in autosomal chromosomes is 3,530 kb (Additional file 1: Figure S1 and Table S1). The number of CNVs estimated for an individual is dependent on array quality and is associated with batch (chemistry plate). In particular, the detection of small CNVs (< 25 kb) requires high quality arrays, whereas identification of large CNVs (> 200 kb) is robust to array quality and batch (Additional file 1: Figure S2). From the distribution of CNV breakpoints across all EA subjects, we identified 12,397 disjoint (non-overlapping) genomic intervals for which copy number is unambiguous and at least 1 percent of ARIC participants have a duplication or deletion (see Methods). These genomic intervals capture 317 non-contiguous loci constituting the CNPs ascertained by the HMM among EA ARIC participants, and nearly all span known regions of copy number variation reported in the Database of Genomic Variants [25].

Prior to our assessment of CNPs as potential risk factors for hyperuricemia, we removed seasonal trends of uric acid concentrations using a lowess smoother with span $\frac{1}{10}$ fit to women and men independently. Our baseline mixed effects model for seasonally adjusted log uric acid concentrations includes fixed effects for study center, age, log BMI, gender, and the interaction of age and log BMI with gender, as well as a random effect for chemistry plate.

For each disjoint interval, we extended the baseline model for uric acid with copy number (0-4) modeled as a continuous covariate. A Manhattan plot of the - log10*p*-value revealed a cluster of statistically significant associations on chromosome 4 (Additional file 1: Figure S3, A). The statistically significant coefficients are derived from two non-overlapping CNPs with NCBI36 build coordinates 9,832,502–9,844,354 bp (CNP-9Mb) and 10,002,240–10,009,754 bp (CNP-10Mb; Additional file 1: Figure S3, B). Together, the two CNPs span 19.368 kb, are interrogated by 49 nonpolymorphic markers and 1 SNP, overlap common deletions previously identified in HapMap Phase 1 [26], and are upstream of the *SLC2A9* gene that is transcribed in the reverse direction. With the exception of the chromosome 4 locus, the distribution of *p*-values is approximately uniform (Additional file 1: Figure S4).

**Figure 1:**
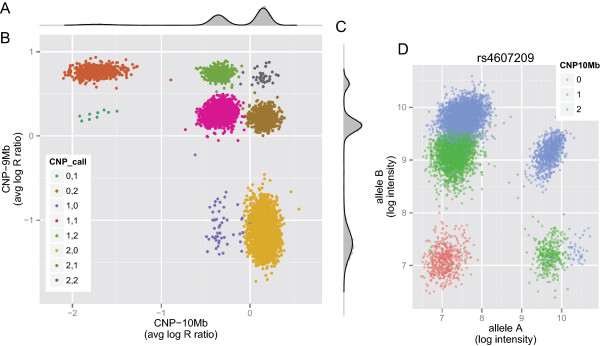
**Low-level data and posterior summaries from a Bayesian finite mixture model supporting copy number alterations. (A)** A histogram of the average log R ratios at CNP-10Mb (gray). The posterior distribution approximated by the Gibbs sampler is indicated by the black lines overlaying the histogram. **(B)** The average log R ratios at the CNP-9Mb and CNP-10Mb chromosome 4 loci. **(C)** Same as **(A)** for the CNP-9Mb locus. **(D)** The log-transformed intensities for alleles A and B allele at a SNP in the CNP-10Mb locus. The genotype clusters are consistent with the copy number estimates from the mixture model.

The marginal distribution of the average log R ratios at CNP-10Mb and CNP-9Mb can be approximated by a mixture of normal distributions, where the components of the mixture are induced by differences in the latent copy number (Figure 1A and 1C). Our approximation to the posterior is derived from a Gibbs’ sampler [27,28], an approach conceptually similar to the Bayesian mixture model described in [29] and extending some of the originally proposed heuristics using mixture models for CNPs [30]. A scatterplot of the average log R ratios at CNP-9Mb and CNP-10Mb provides a non-discrete visualization of their joint distribution (Figure 1B). Assuming the mixture components correspond to latent copy numbers 0, 1, and 2, the integer copy number for each sample is inferred from the component with highest posterior probability. The copy number estimates from the mixture model are further corroborated by the genotype clusters for SNP rs4607209 in the CNP-10 Mb locus (Figure 1D). For example, samples belonging to the second mixture component (copy number 1) populate the 'A’ and 'B’ genotype clusters at SNP rs4607209 (green). Hereafter, regression models for uric acid utilize the maximum a posteriori copy number estimates from the Bayesian mixture model.

**Figure 2:**
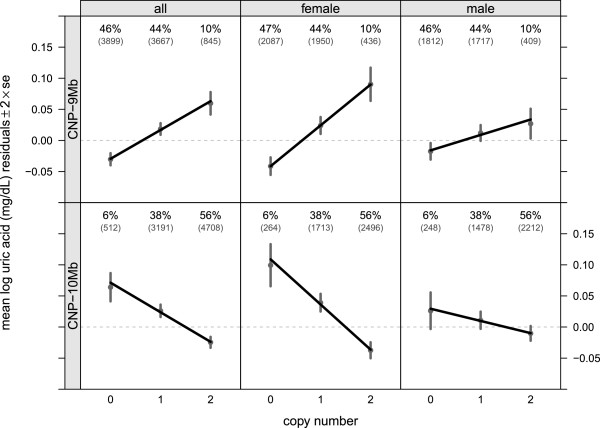
**The relationship between integer copy number (x-axis) and average log uric acid concentrations is approximately linear.** Slopes for the copy number coefficients at the chromosome 4 CNP-9 Mb (top) and CNP-10 Mb (bottom) loci overlay the empirical average log uric acid concentration with error bars drown to ± two standard errors of the mean. The opposite signs of the regression slopes at CNP-9Mb and CNP-10Mb is a reflection of linkage disequilibrium – the copy number estimates have a strong, negative correlation (Spearman correlation = -0.82).

Copy number estimates at the CNP-9 Mb and CNP-10 Mb loci have a Spearman correlation coefficient of -0.82. Homozygous deletions are common at each locus (46% of subjects at the CNP-9Mb locus and 6% of subjects at the CNP-10Mb locus), yet none of the subjects have a homozygous deletion at both loci (233 expected by chance). Evaluated in separate regression models, each deleted copy at CNP-9Mb and CNP-10Mb is associated with a _1.17_1.50_1.82_ percentage decrease (*p*=5.43×10^-20^) and a _1.83_2.63_3.42_ percentage increase (*p*=1.54×10^-10^) in uric acid concentrations, respectively (Figure 2). While the regression coefficients at CNP-9Mb and CNP-10Mb are opposite in sign, the data is consistent with a dose response to copy number at only one CNP and an opposing sign for the tagging CNP attributable to its strong linkage disequilibrium. At each locus, the interaction of copy number and gender is statistically significant with more pronounced slopes observed among women. For example, each deleted copy at the CNP-10 Mb CNP among women is associated with a _3.97_4.93_5.87_ (*p*=4.57×10^-23^) percentage increase of uric acid concentrations, whereas among men each deleted copy is associated with a _0.31_1.36_2.39_ (*p* = 0.001) percentage increase in uric acid concentrations.

**Figure 3:**
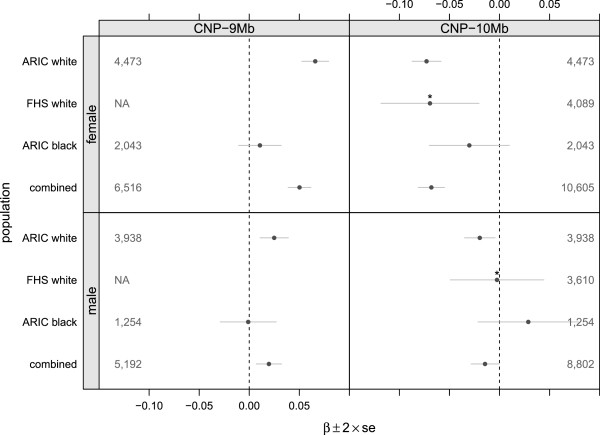
**Regression coefficients for copy number at the CNP-9 Mb and CNP-10 Mb loci in ARIC and FHS cohorts.** Combined estimates were obtained by a weighted average using the inverse variance of the model coefficients as weights. Data is not available at the CNP-9 Mb loci in FHS due to the older array technology. ^∗^Missing genotypes at SNP rs4607209 in the CNP-10 Mb locus are modeled as a surrogate for deletion genotypes in FHS.

To evaluate whether CNPs at the chromosome 4 loci are associated with uric acid in an independently sampled EA population for which uric acid measurements are available, we pursued replication in FHS. Because access to the intensity-level data in FHS was not available, we used missing genotype calls for SNP rs4607209 in the CNP-10 Mb CNP as a surrogate for the deletion polymorphism (justification in Methods). With the missing genotype indicator as a surrogate for homozygous deletions, we fit a mixed effects model implemented in the R package kinship [31] with log uric acid concentrations as the dependent variable and clinical covariates age, gender, and log-transformed BMI as explanatory variables. The gender-specific slopes for the surrogate copy number variable in FHS are comparable to the copy number slopes in ARIC with respect to magnitude, direction, and statistical significance (Figure 3). In particular, missing genotypes are associated with a _1.41_4.70_7.88_ percentage increase of uric acid concentrations among FHS women (*p*=5.46×10^-03^) compared to a _3.97_4.93_5.87_ percentage increase among ARIC women (*p*=4.57×10^-23^). As in ARIC, the _-3.12_0.17_3.36_ percentage change in uric acid concentrations among men is small and not statistically significant in FHS (*p*=0.92). Replication at the CNP-9 Mb CNP is not possible as the array platform used in FHS does not contain markers in this region.

To investigate whether the association between copy number and uric acid concentrations is present in non-EA populations, we estimated the copy number at both chromosome 4 CNPs for 3,392 African American (AA) participants in ARIC using the Bayesian mixture model described previously for the EA cohort. Homozygous deletions occur in approximately 46 and 6% of EA participants at the CNP-9Mb and CNP-10 Mb loci, respectively, but only 33 and 0.6% of AA participants have homozygous deletions at these loci. The percentage decrease of uric acid concentrations associated with each deleted copy at CNP-9 Mb is _-0.75_0.73_2.22_ among women (*p*=0.335) and _-1.90_0.05_1.97_ among men (*p*=0.957). Similarly, copy number is not associated with uric acid levels among AA women or men at CNP-10 Mb ($\chi_{2\text{df}}^{2}=3.45,p=0.179$) (Figure 3).

**Figure 4:**
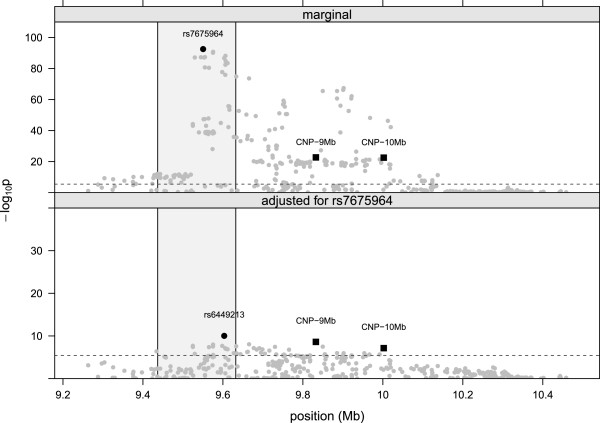
**SNP and CNP associations near *SLC2A9 *with and without adjustment for genome-wide significant SNP rs7675964.** Top: Negative log10*p*-values derived from a likelihood ratio test comparing a null model with clinical and technical covariates to an extended model evaluating the marginal association of SNPs (gray circles) or CNP×gender (black rectangles). The region shaded in light gray is the location of the *SLC2A9* gene. Bottom: Negative log10*p*-values from a likelihood ratio test comparing an extended model with SNPs or CNP × gender to a null model that includes the rs7675964 genotypes.

To assess whether the CNP associations are independent of *SLC2A9* SNPs among EA participants, we evaluated a series of models for uric acid concentrations that include SNPs and/or the gender-specific CNP slopes. Marginally, the association between SNPs and CNPs with uric acid concentrations is the strongest for SNPs directly in the *SLC2A9* transcript, and the associations 200 kb upstream of *SLC2A9* are comparable for SNPs and CNPs (Figure 4, top). Adjusting for the SNP with the strongest marginal association (rs7675964), effect sizes for other SNPs near *SLC2A9* decrease. The CNP effect sizes are also attenuated but remain genome-wide significant (minimum $\chi_{2\text{df}}^{2}=3190,\;p=7.23\times 10^{-08}$) (Figure 4, bottom). Adjusted for the CNP with the strongest marginal association (CNP-9 Mb), the effect size for SNP rs7675964 is comparable to the marginal model (data not shown).

**Figure 5:**
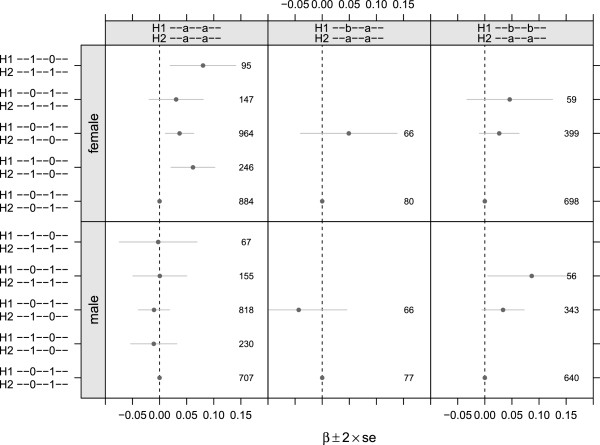
**The association of CNP haplotypes with uric acid levels is independent of genome-wide significant SNPs.** Genotypes at rs7675964 and rs6449213 were phased with CNP-9 Mb and CNP-10 Mb. Subjects were stratified into three allelic haplotypes (column labels) for which there was variation in the CNP haplotypes (y-axis labels). The pair of CNP haplotypes given by ${}_{\text{H2:}--0--1--}^{\text{H1:}--0--1--}$ is the reference group for each regression. Likelihood ratio tests for the CNP haplotypes are statistically significant for women with allelic haplotypes ${}_{\text{H2:}--a--a--}^{\text{H1:}--a--a--}$ ($\chi_{4\text{df}}^{2}=14.3,\;p=6.3\times 10^{-03}$) and marginally significant for both men and women with allelic haplotypes ${}_{\text{H2:}--a--a--}^{\text{H1:}--b--b--}$ ($\chi_{4\text{df}}^{2}=9.05,\;p=0.0599$). CNP haplotypes are not associated with uric acid concentrations among ${}_{\text{H2:}--a--a--}^{\text{H1:}--b--a--}$ subjects ($\chi_{2\text{df}}^{2}=2.06,\;p=0.357$), though the sample size for this cohort is small and the effect size among the 66 women is comparable to the effect size in the much larger ${}_{\text{H2:}--a--a--}^{\text{H1:}--b--b--}$ and ${}_{\text{H2:}--a--a--}^{\text{H1:}--a--a--}$ subgroups.

While regression coefficients for SNPs near *SLC2A9* are attenuated in the rs7675964-adjusted models, SNP rs6449213 (and others) remain genome-wide significant (*p*=9.46×10^-11^). To assess the independence of the CNP association with uric acid after adjusting for the rs6449213 and rs7675964 genotypes, we compared the baseline mixed effects model with rs6449213 and rs7675964 genotypes to an extended model with gender-specific slopes for copy number. A 2 degree of freedom likelihood ratio test comparing the baseline and extended models is statistically significant at both CNP loci (CNP-9 Mb:$\chi_{2\text{df}}^{2}=31,p=2.01\times 10^{-07}$; CNP-10 Mb: $\chi_{2\text{df}}^{2}=33,p=8.72\times 10^{-08}$). To further evaluate whether CNPs contribute to inter-individual variation of uric acid concentrations independently of SNPs in *SLC2A9*, we phased the genotypes at rs7675964 and rs6449213 with copy number at CNP-9 Mb and CNP-10 Mb (see Methods). Notationally, we denote the CNP portion of the haplotypes by $\begin{array}{c} \text{H1:}--c_{1,1}--c_{1,2}--\\ \text{H2:}--c_{2,1}--c_{2,2}-- \end{array}$, where *c*_*i*,*j*_ is the copy number at the *j*^*t* *h*^ CNP locus (*c*_*i*,*j*_∈{0,1}) for haplotype *H*_*i*_ (*i*∈{1,2}). Similarly, the portion of the haplotypes for rs7675964 and rs6449213 are denoted by $\begin{array}{c} \text{H1:}--g_{1,1}--g_{1,2}--\\ \text{H2:}--g_{2,1}--g_{2,2}-- \end{array}$,where *g*_*i*,*j*_ is the allele at the *j*^*t* *h*^ SNP (*g*_*i*,*j*_∈{*a*,*b*}). Of the 2^4^ possible allelic haplotypes, 14 were observed in the 8,411 EA participants and only 3 SNP haplotypes had variation in the corresponding CNP haplotype. Specifically, the 3 SNP haplotypes for we observed variation in the phased copy number estimates are $\begin{array}{c} \text{H1:}--a--a--\\ \text{H2:}--a--a-- \end{array}$, $\begin{array}{c} \text{H1:}--b--b--\\ \text{H2:}--a--a-- \end{array}$,and $\begin{array}{c} \text{H1:}--b--a--\\ \text{H2:}--a--a-- \end{array}$. For 2,195 subjects with the allelic haplotype $\begin{array}{c} \text{H1:}--b--b--\\ \text{H2:}--a--a-- \end{array}$, CNP haplotypes $\begin{array}{c} \text{H1:}--0--1--\\ \text{H2:}--1--0-- \end{array}$ and $\begin{array}{c} \text{H1:}--0--1--\\ \text{H2:}--1--1-- \end{array}$ are weakly associated with uric acid concentrations with similar effect sizes observed in men and women ($\chi_{4\text{df}}^{2}=9.05,\;p=0.0599$). For 4,313$\begin{array}{c} \text{H1:}--a--a--\\ \text{H2:}--a--a-- \end{array}$ subjects, CNP haplotypes are associated with uric acid concentrations in women ($\chi_{4\text{df}}^{2}=14.3,\;p=6.3\times 10^{-03}$) but not men ($\chi_{4\text{df}}^{2}=0.757,\;p=0.944$). CNP haplotypes are not associated with uric acid concentrations for $\begin{array}{c} \text{H1:}--b--a--\\ \text{H2:}--a--a-- \end{array}$subjects ($\chi_{2\text{df}}^{2}=2.06,\;p=0.357$), though the sample size for this population is small and the effect size among the 66 women in this subgroup is comparable to the effect size in the much larger $\begin{array}{c} \text{H1:}--b--b--\\ \text{H2:}--a--a-- \end{array}$ and $\begin{array}{c} \text{H1:}--a--a--\\ \text{H2:}--a--a-- \end{array}$ subgroups for which the CNP haplotype association is statistically significant (Figure 5).

As the CNP association appears independent of *SLC2A9* SNPs and the CNP loci are located in an intergenic region approximately 200 kb upstream of the *SLC2A9* gene (*SLC2A9* is transcribed in the reverse orientation), we examined publicly available regulatory data for human kidney tissue where *SLC2A9* is known to function in the transport of uric acid from urine to blood [32]. Examination of DNAse hypersensitivity for human fetal kidney tissue and adult kidney cell line HKC8 revealed a peak adjacent to CNP-10 Mb, suggesting that CNP-10 Mb abuts a regulatory element. We did not observe DNAse hypersensitivity peaks near CNP-9 Mb, but nearly half of EA participants have a homozygous deletion at CNP-9 Mb. It is unclear whether the absence of peaks at CNP-9 Mb reflect the absence of a regulatory element in the fetal kidney or whether the fetal kidney has a deletion at this locus (i.e., loss of a regulatory element by deletion).

Given the strong association between CNPs and uric acid, we modeled the relationship between CNPs and gout. Of the 8,411 ARIC EA participants, 609 had gout at some point during the study’s follow-up. In a logistic regression model including technical and clinical covariates described previously, the odds of gout is 1.21 times higher comparing subjects who differ by one copy of CNP-9 Mb (*p*=0.003). As expected, this association is largely mediated through the CNP’s association with serum uric acid. After including uric acid in the model, the association between copy number at CNP-9 Mb and gout is attenuated (1.11 odds ratio; p=0.12). Results are qualitatively similar at the CNP-10 Mb locus with a statistically significant gout association in the marginal model that is attenuated after adjusting for uric acid concentrations (data not shown).

## Conclusions

This study is the first genome-wide scan of CNPs and uric acid. We identified an association between serum uric acid concentrations and two common, intergenic deletions that are 200 kb and 350 kb, respectively, upstream of the urate transporter *SLC2A9*. Loss of DNA copy number in these regions is associated with ≈5 percent change of uric acid concentrations among women and a one percent change among men with the direction of the effect depending on the CNP locus ($\chi_{2\text{df}}^{2}=3545$, *p*=3.19×10^-23^). Gender-specific associations between *SLC2A9* polymorphisms and uric acid concentrations have been reported by others and are consistent with our observations with CNPs near *SLC2A9*[7,33-36]. Independent replication of the association between copy number and uric acid concentrations in FHS provides further support for our finding. Among ARIC AA participants, CNP-10 Mb is weakly associated with uric acid concentrations and there was no association at CNP-9 Mb in men or women. The CNP association in ARIC EA is independent of previously reported SNP associations in *SLC2A9*, as assessed by joint CNP and SNP regression models as well as regression models with phased SNP and CNP haplotypes.

The physiological role of *SLC2A9* in the kidney is the reabsorption of urate from urine into blood, leading to increased levels of serum uric acid concentrations when *SLC2A9* expression is up-regulated and decreased levels with loss of function mutations such as deletions. When phased with genome-wide significant SNPs in *SLC2A9*, the haplotypes with homozygous deletions at CNP-9 Mb had lower uric acid concentrations as we would hypothesize if CNP-9 Mb spans an enhancer for *SLC2A9*. DNAse hypersensitivity assays suggest that CNP-10 Mb abuts a regulatory element, but we did not find DNAse hypersensitivity or ChiP-seq peaks at CNP-9 Mb. Assays from other cell lines in ENCODE are consistent with our findings in the kidney. For example, CNP-10 Mb spans DNAse hypersensitivity peaks in normal esophageal epithelial cells (HEEpiC cell line), airway epithelial cells (SAEC cell line), epidermal keratinocytes (cell line NHEK), and mammary epithelial cells (HMEC cell line), as well as a H3KMe1 histone mark in HMEC cells [37]. As nearly 50 percent of EA participants in ARIC have homozygous deletions at CNP-9 Mb, it is possible that the fetal kidney cell line harbors a homozygous deletion at this locus and that the absence of ChiP-seq binding and DNAse hypersensitivity reflect absence of regulatory elements due to loss of DNA copy number. Gene expression data for kidney or liver tissues and germline copy number for the same samples is not currently available in ARIC or FHS.

Our CNP GWAS has low sensitivity for deletions less than 50 kb in size and/or having fewer than 10 Affymetrix 6.0 markers. For amplifications, the inability to discriminate high copy amplifications from single- and two- copy duplications because of the limited dynamic range of the array platform will attenuate the regression coefficients for copy number. The attenuation of the copy number coefficients for amplifications occurs irrespective of the size of the amplicon, but will be worse for small, focal amplifications due to the limited resolution of the platform. Our analyses do not rule out the contribution of small insertions and deletions as well as high copy repeats that are beyond the dynamic range of high-throughput arrays. Sequencing platforms will be useful for elucidating whether additional structural and mutational variants near *SLC2A9* contribute to inter-individual heterogeneity of uric acid concentrations. In addition, our association analysis only included CNPs. Rare duplications and deletions such as those directly spanning the *SLC2A9* transcript (5 deletions and 9 duplications in ARIC) were not evaluated in our analysis of CNPs and may have a larger effect on uric acid concentrations than the CNPs studied here. While these limitations impact sensitivity, our results indicate that CNP genome-wide association studies can achieve a high degree of specificity. As in any high-throughput setting, the specificity of a genome-wide screen depends on the extent to which technical factors influencing estimation can be modeled and the degree to which they are independent of the outcome of interest. Participants in ARIC were neither enrolled nor processed on the basis of their uric acid concentrations. Due to the merits of the experimental design and mixed models for uric acid that adjust for study center and chemistry plate, we feel the major sources of artefactual associations in ARIC have been addressed.

In summary, the loss of several kilobases of DNA in close proximity to *SLC2A9*, a known uric acid transporter and a candidate gene for gout [38-40], presents a biologically plausible mechanism for regulation of *SLC2A9* expression and modulation of serum uric acid concentrations. Gene expression data on the same set of individuals in target kidney and liver tissues is needed to evaluate whether loss of DNA copy number effects transcription of *SLC2A9* as hypothesized, and to evaluate gender differences in *SLC2A9* expression.

## Methods

This paper follows the guidelines for communicating confidence intervals as suggested in [41]. Institutional Review Board (IRB) approval was obtained by the Johns Hopkins University ARIC study center, and the research was conducted in accordance with the principles described in the Declaration of Helsinki.

### ARIC study

The ARIC study is an ongoing, prospective community-based cohort of 15,792 persons (27% black) aged 45-64 years at baseline (1987-89) [42]. Participants were selected by probability sampling from four U.S. communities (Forsyth County, North Carolina; Jackson, Mississippi; Minneapolis, Minnesota; and Washington County, Maryland). Participants took part in examinations starting with a baseline visit between 1987 and 1989 and three follow-up visits, thereafter, administered three years apart (visit 2: 1990-1992; visit 3: 1993-1995; visit 4: 1996-1998). At baseline, a home interview assessed participants’ sociodemographic characteristics, smoking, and alcohol-drinking habits, medication use, and medical history. A clinical examination included measurement of various risk factors. All participants self-reported race as Asian, black, American Indian, or white. Body-mass index (BMI) was measured according to published methods [43]. Central laboratories performed analyses on baseline fasting specimens using conventional assays to obtain uric acid values [44]. Uric acid was measured by the uricase method [45]. The reliability coefficient of uric acid was 0.91, and within-person variability was 7.2 [46].

### CNV estimation

Raw CEL files from scanned Affymetrix 6.0 arrays were processed using Affymetrix power tools (APT, version 1.14.3) and PennCNV to derive estimates of log R ratios and B allele frequencies at each marker. While the log R ratio estimates were wave-adjusted [21], genomic waves persisted in many of the ARIC samples. We further processed the log R ratios using the R package ArrayTV [47] – an approach adapted from software for removing waves in high-throughput sequencing data [48]. A 6-state HMM comprising 5 distinct copy number states (0-4) implemented in the R package VanillaICE (VI) and the stand-alone tool PennCNV were applied independently to each sample [13,14,49]. CNVs with fewer than 10 markers were excluded due to the level of noise of the log R ratios and the difficulty in assessing the validity of low-coverage CNVs without experimental validation. As inference from association models using the PennCNV- and VI- derived copy number estimates were found to be qualitatively similar, only the *VI* copy number associations were reported.

### Quality control measures

Among 9,779 samples of EA for whom uric acid concentrations were measured at visit 1, we excluded 743 samples that did not meet criteria for SNP genome-wide association analyses in ARIC as described in Köttgen *et al.*[50]. For the estimation of germline CNVs, high CNV call frequencies often indicate problems with the normalization such as genomic waves that were incompletely removed by the wave correction methods. We excluded 625 participants with autosomal log R ratios having high autocorrelation or variance (lag 10 autocorrelation > 0.03 or median absolute deviation > 0.32), or if the number of CNVs called by the VI algorithm exceeded 100. We used the signal to noise ratio (SNR) implemented in the R package crlmm as a sample-specific measure of array quality as assessed by the overall separation of the canonical genotype clusters at SNPs [51,52], but we did not exclude samples on the basis of this statistic. Following the above quality control filters, 8,411 EA participants were evaluated in the subsequent association models.

### Genome-wide scan of copy number and uric acid levels

From the set of genomic intervals defining CNVs derived by the VI HMM fit to 8,411 EA subjects, we constructed rectangular matrices of the inferred integer copy number. Element [*i*,*j*] of the matrix is the copy number at genomic interval *i* for sample *j*. The genomic intervals were obtained from the union of the start and end coordinates across all CNVs detected for each of the autosomal chromosomes with the requirement that each non-overlapping (disjoint) interval contain at least one marker. For each disjoint interval, we calculated the number of samples harboring a CNV, excluding intervals for which fewer than one percent of the samples had a CNV. Across samples, the CNVs are partially overlapping and any given CNV may span one or many disjoint intervals. As a consequence, adjacent disjoint intervals often convey similar information with comparable frequencies of deletions and duplications. As the test statistics are correlated, Bonferonni correction is conservative. Because none of the loci were of borderline statistical significance (Additional file 1: Figure S3), more sophisticated simulation-based approaches for multiple testing correction with dependent test statistics were not assessed.

Mixed effects regression models for ARIC cohorts were implemented using the R package lme4 [53]. Specifically, we modeled seasonally adjusted serum log uric acid concentrations (continuous) in a regression model with fixed effects for copy number (modeled as continuous with scale 0-4), age (continuous), log-transformed BMI (continuous), gender, and study center (categorical). As the heavy-tailed uric acid concentrations were log-transformed, we report the percentage change of uric acid concentrations per integer increase in copy number. To take into account the heterogeneity of CNV call frequencies between chemistry plates, we include chemistry plate as a random effect. For regression models with canonical genotypes as covariates, we treated the frequency of the B-allele (an integer in the set 0, 1, or 2) as continuous. For FHS, we implemented mixed effects regression models using the R package kinship (http://cran.uvigo.es/src/contrib/Archive/kinship/) [31].

### Imputation of copy number in the Framingham heart study

To evaluate whether CNPs at the chromosome 4 loci are associated with uric acid in an independently sampled EA population, we explored replication in FHS. Challenges to replication in FHS include the older array architecture (Affymetrix 250k Nsp/Sty chips) and the unavailability of raw intensities needed for copy number estimation. While there were no markers for CNP-9 Mb on the 250k chips, SNP rs4607209 in CNP-10 Mb is present in the Affymetrix 250k Nsp chip. To verify that the expected non-diploid genotypes ('A’, 'B’, and NULL genotypes) can be observed from the normalized intensities for this SNP on the Affymetrix 250k Nsp chip, we genotyped the 270 phase 2 HapMap samples that were assayed on the the Affymetrix 250k platform using the BRLMM algorithm implemented in Affymetrix power tools. (The BRLMM algorithm was used to genotype FHS participants.) A scatterplot of the log intensities for the A and B alleles reveals three clusters corresponding to the deletion genotypes for rs4607209 in addition to the canonical biallelic clusters (Additional file 1: Figure S5), and is similar to the clusters observed on the Affymetrix 6.0 platform for ARIC EA participants (Figure 1D). Homozygous deletions occur in 8.9% of the HapMap CEPH samples and 6.1% of the ARIC EA participants. The canonical biallelic genotypes in HapMap have high genotype confidence scores (not shown) and no missing calls, while 6 out of 8 CEPH subjects with homozygous deletions have missing BRLMM genotype calls. These data demonstrate that the low level intensities for SNP rs4607209 in both the 250k Nsp and Affymetrix 6.0 platforms have distinct clusters corresponding to the latent copy number and that missing BRLMM genotypes occur in clusters that are consistent with homozygous deletions. The specificity of missing genotype calls as a surrogate for homozygous deletion genotypes at SNP rs4607209 in EA HapMap is 1 and the sensitivity is 0.75. We expect that missing genotype calls as a surrogate for homozygous deletions will lead to conservative parameter estimates of the copy number effect size in regression models as contamination of the diploid population with subjects harboring homozygous and hemizygous deletions will bias the regression slopes to zero.

### Estimation of copy number for ARIC AA participants

Log R ratios for markers in the CNP-9 Mb and CNP-10 Mb loci were averaged. The average log R ratios in AA participants are a mixture of 3 normal distributions as observed in the EA population, with the mixture components presumed to be induced by differences in the latent copy number. A Gibbs’ sampler [27,28] was implemented in R to approximate the posterior distribution of the 3-component normal mixture. Each subject was assigned to the mixture component with the highest posterior probability. As in the EA cohort, the observed mixture components in the AA cohort are most consistent with homozygous deletion, hemizygous deletion, and diploid copy number on the basis of the expected log R ratios for these copy number states.

### Phasing SNPs and CNPs near *SLC2A9*

Genotypes from 8 SNPs having the largest marginal associations with uric acid (including rs7675964 and rs6449213) were phased with CNP-9 Mb and CNP-10 Mb using the fastPHASE software [54]. For diploid CNPs, we assumed that each haplotype had one copy. This assumption is supported empirically by the data–if haplotypes containing two copies were common, we would expect to see subjects with duplications. Haplotypes were modeled as categorical covariates in regression models for uric acid concentrations. Subjects with rare haplotypes and subjects with allelic haplotypes that had no variation in the corresponding CNP portion of the haplotypes were excluded (1,473 subjects).

### Genomic annotation and software versions

Genomic annotation in this paper is based on UCSC build hg18 (NCBI36) [55]. Gene *SLC2A9* has RefSeq accession numbers NM_001001290.1 and NM_020041.2. We used the May, 2010 version of PennCNV, version 1.14.3 of APT, and version 1.4.0 of fastPHASE [54]. All remaining analyses were performed in the statistical environment R[56]. Graphics were generated using the R packages lattice [57] or ggbio [58,59]. The analyses downstream of the VI algorithm relied on the infrastructure provided by the GenomicRanges package [60]. The complete listing of supporting R packages and their corresponding version numbers is provided below. 

•R version 3.1.0 (2014-04-10), x86_64-apple-darwin13.1.0

•Base packages: base, datasets, graphics, grDevices, grid, methods, parallel, stats, tools, utils

•Other packages: aricUricAcid 1.0.19, Biobase 2.24.0, BiocGenerics 0.10.0, Biostrings 2.32.0, DBI 0.2-7, devtools 1.5, foreach 1.4.2, GenomeInfoDb 1.0.2, GenomicRanges 1.16.3, ggplot2 1.0.0, gridExtra 0.9.1, gtable 0.1.2, IRanges 1.22.7, knitr 1.6, lattice 0.20-29, lme4 1.1-6, Matrix 1.1-3, oligo 1.28.2, oligoClasses 1.26.0, pd.genomewidesnp.6 1.10.0, RColorBrewer 1.0-5, Rcpp 0.11.1, RSQLite 0.11.4, XVector 0.4.0

•Loaded via a namespace (and not attached): affxparser 1.36.0, affyio 1.32.0, BiocInstaller 1.14.2, bit 1.1-12, codetools 0.2-8, colorspace 1.2-4, digest 0.6.4, evaluate 0.5.5, ff 2.2-13, formatR 0.10, gtools 3.4.0, httr 0.3, iterators 1.0.7, latticeExtra 0.6-26, MASS 7.3-33, memoise 0.2.1, minqa 1.2.3, munsell 0.4.2, nlme 3.1-117, plyr 1.8.1, preprocessCore 1.26.1, proto 0.3-10, RcppEigen 0.3.2.1.2, RCurl 1.95-4.1, reshape2 1.4, scales 0.2.4, splines 3.1.0, stats4 3.1.0, stringr 0.6.2, whisker 0.3-2, zlibbioc 1.10.0

## Availability of supporting data

The data set supporting the results of this article is available in the dbGaP repository, phs000090.v1.p1 (http://www.ncbi.nlm.nih.gov/projects/gap/cgi-bin/study. cgi?study_id=phs000090.v1.p1). The ChiP-seq and DNAase hypersensitivity data for the kidney described in [32] is available from the GEO repository, accession: GSE49637 (http://www.ncbi.nlm.nih.gov/geo/query/acc.cgi?acc=GSE49637).

## Abbreviations

AA: African American; ARIC: Atherosclerosis risk in communities; BMI: Body mass index; ChiP: Chromatin immunoprecipitation; CNP: Copy number variant; CNP: Copy number polymorphism; EA: European ancestry; FHS: Framingham heart study; HMM: Hidden Markov model; MAD: Median absolute deviation; SNP: Single nucleotide polymorphism; SNR: Signal to noise ratio.

## Competing interests

The authors declare that they have no competing interests.

## Authors’ contributions

RBS, JC, and WKHL conceived of the study. RBS, LM, AK, EB, CSF, AC, KS, and WKHL drafted the manuscript. KS and AK participated in the analysis and interpretation of DNAse hypersensitivity and ChIP-seq assays. RBS, LM, EHS, QY, IR, AT, and SC participated in the statistical analyses. All authors read and approved the final manuscript.

## Supplementary Material

Additional file 1 **Supplementary figures and tables.Figure S1:** Size, frequency and burden of CNVs among ARIC participants of European ancestry. **Figure S2:** Batch effects in processing arrays for copy number estimation. **Figure S3:** Manhattan plot of copy number associations. **Figure S4:** Quantile-quantile plot of the expected -log10*p*-values versus the observed -log10*p*-values. **Figure S5:** A scatterplot of the normalized intensities for the A and B alleles of SNP rs4607209 for 90 HapMap subjects of EA assayed on the Affymetrix 250k Nsp chip used in FHS. **Table S1:** Median and interquartile range (IQR) descriptive statistics of CNVs for 8,411 EA participants. Click here for file
