# Supplementary material for: Copy number polymorphisms near SLC2A9 are associated with serum uric acid concentrations
Source: BMC Genet. 2014 Jul 9;15:81. doi: 10.1186/1471-2156-15-81 (PMC4118309; doi:10.1186/1471-2156-15-81)
Supplement: Additional file 1 — Supplementary figures and tables.Figure S1: Size, frequency and burden of CNVs among ARIC participants of European ancestry. Figure S2: Batch effects in processing arrays for copy number estimation. Figure S3: Manhattan plot of copy number associations. Figure S4: Quantile-quantile plot of the expected -log10p-values versus the observed -log10p-values. Figure S5: A scatterplot of the normalized intensities for the A and B alleles of SNP rs4607209 for 90 HapMap subjects of EA assayed on the Affymetrix 250k Nsp chip used in FHS. Table S1: Median and interquartile range (IQR) descriptive statistics of CNVs for 8,411 EA participants. [file 1471-2156-15-81-S1.pdf]

Additional file 1: supplementary figures and table

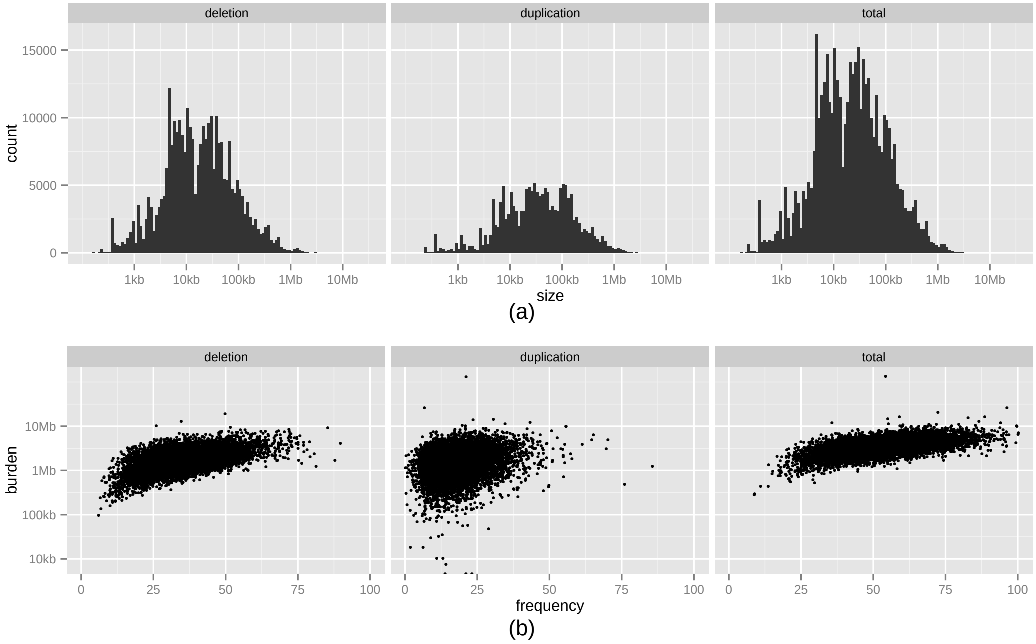

Figure S1 Size, frequency and burden of CNVs among ARIC participants of European ancestry. CNVs were estimated using the VI algorithm. (Top): Size of CNVs stratified by qualitative categories of copy number loss or gain. (Bottom) Frequency of CNVs per individual plotted against the total number of basepairs in CNVs (burden).

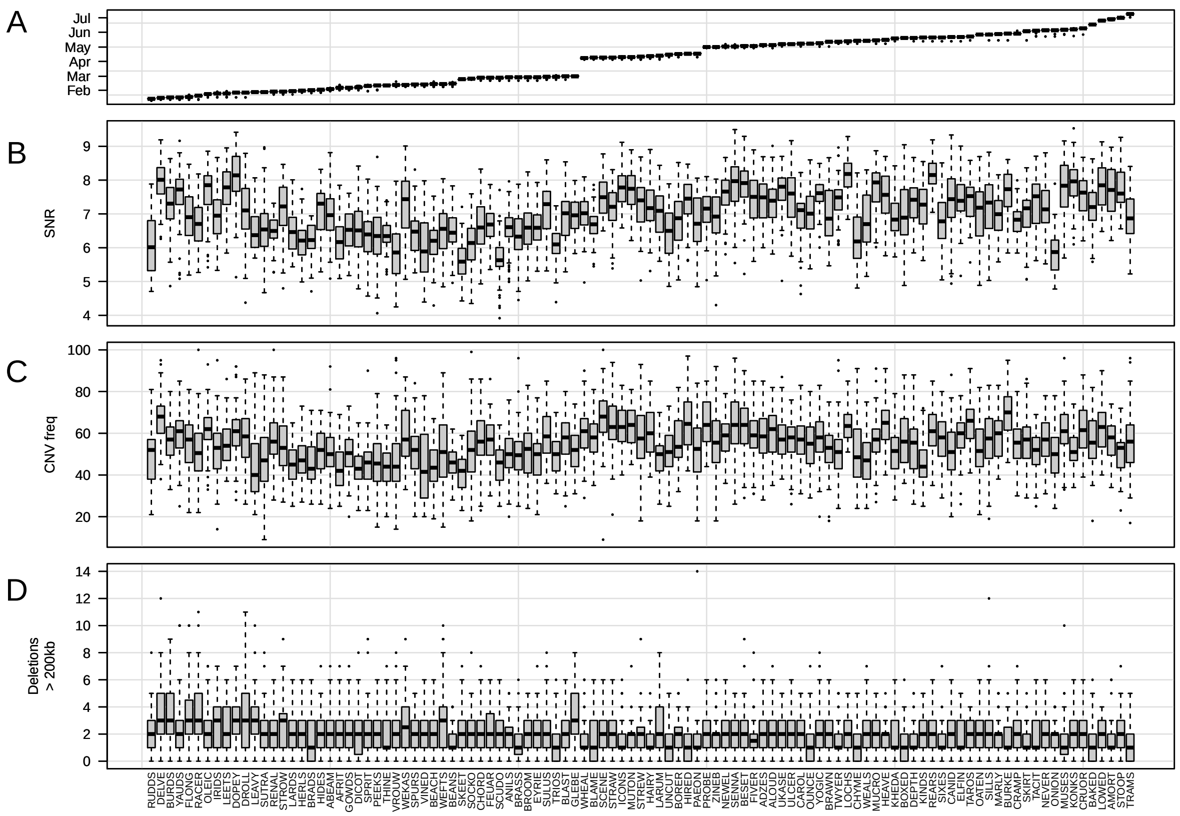

Figure S2 Batch effects on low-level summaries. (A) The distribution of the array scan dates (y-axis) stratified by chemistry plate. Plates are ordered by the median scan date, and only plates with 50 or more samples are displayed. (B) The distribution of the signal to noise ratio (SNR), a measure of array quality, stratified by plate. (C) Plates with higher SNRs have more CNV calls as a result of the increased resolution. (D) The detection of deletions greater than 200 kb are robust to differences in array quality.

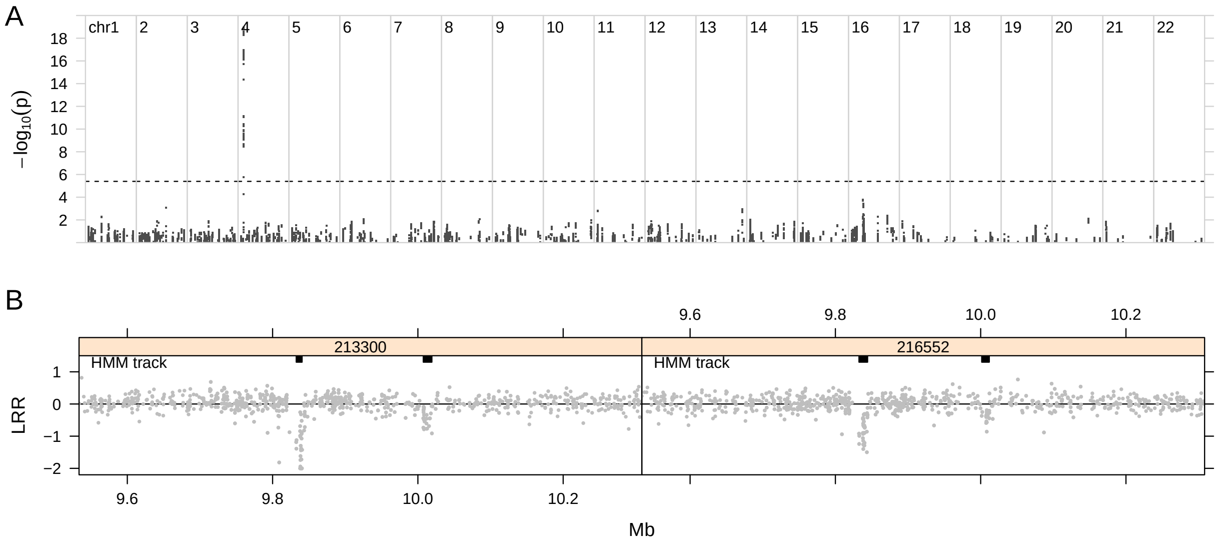

Figure S3 Manhattan plot of copy number associations. (A) Genome-wide association between copy number and serum uric acid concentrations adjusted for clinical and technical covariates. A conservative estimate for genome-wide statistical significance as assessed by Bonferroni correction is indicated by the horizontal dashed line. (B) The signal at chromosome 4 can be resolved from two non-overlapping CNPs with genomic coordinates 9,832,502–9,844,354 and 10,002,240–10,009,754 bp. For example, sample 213300 (left) and sample 216552 (right) each have deletions at the two chromosome. 4 CNPs. A track indicating the hemizygous deletions called by the HMM are plotted as black rectangles.

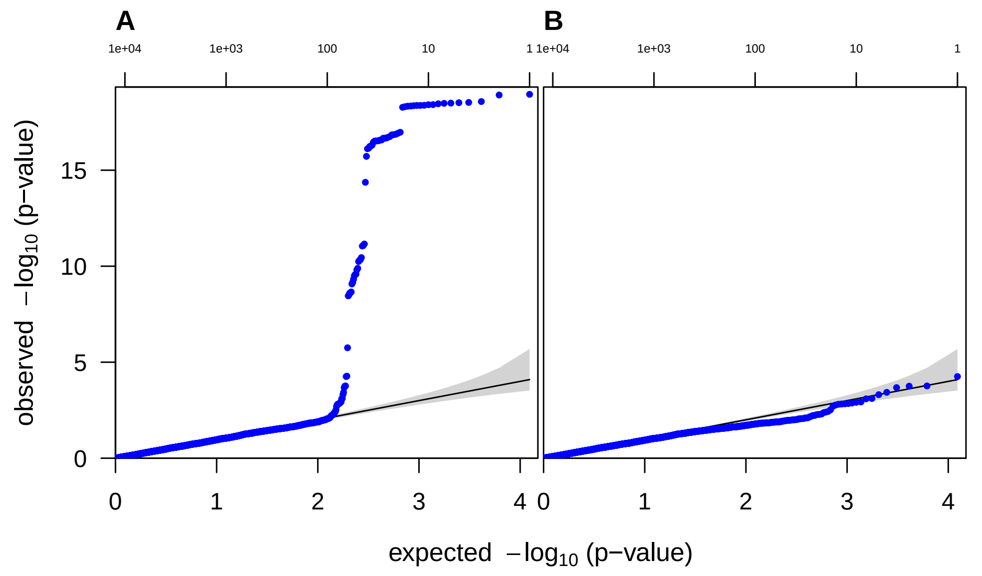

Figure S4 Quantile-quantile plot of the expected negative $\log_{10}$ p-values versus the observed negative $\log_{10}$ p-values with (panel A) and without (panel B) markers in the two CNP loci on chromosome 4. The x-axes drawn on the top margins denotes the p-value rank.

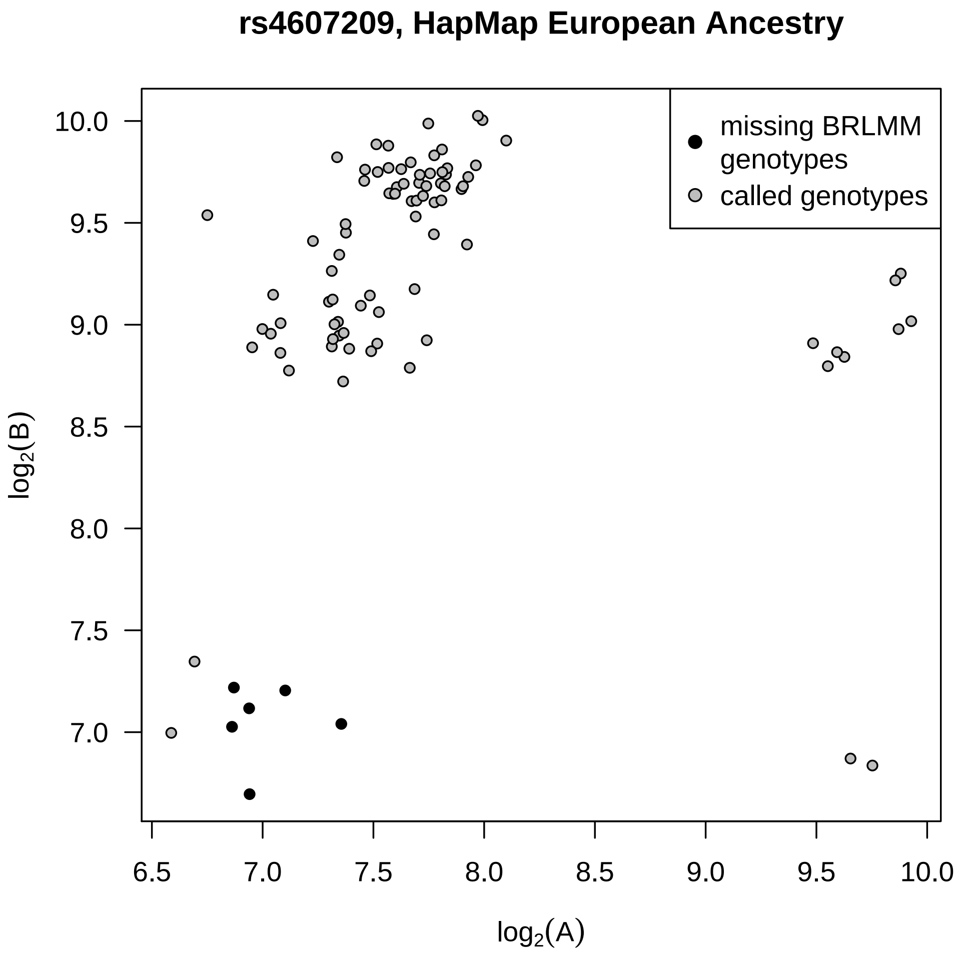

Figure S5 A scatterplot of the normalized intensities for the A and B alleles of SNP rs4607209 for 90 HapMap subjects of EA assayed on the chip used by FHS (Affymetrix 250k Nsp). Homozygous deletions occur in 8.9% of the HapMap subjects and 6.1% of the ARIC EA participants. Missing genotype calls at this SNP have a specificity of 1 for homozygous deletions and a sensitivity of 0.75.

Supplementary Tables

	overall (IQR)	deletions (IQR)	duplications (IQR)
size [kb]	22 (18, 27)	22 (18, 27)	36 (26, 51)
bases altered [kb]	3530 (2695, 4491)	1701 (1191, 2433)	1508 (903, 2332)
frequency	55 (45, 64)	36 (28, 43)	17 (13, 23)
number genes	45 (35, 55)	24 (18, 31)	24 (18, 31)

Table S1 Median and interquartile range (IQR) descriptive statistics of autosomal CNVs identified in 8,411 EA participants. The number of genes is tabulated differently for deletions and duplications. For deletions, a gene is counted if any portion of the gene overlaps a deletion. For duplications, a gene is counted only if the entire transcript is spanned by an amplification.
